# Precarious work at a surgical center: implications for the organization and for the health of nursing workers

**DOI:** 10.1590/0034-7167-2022-0120

**Published:** 2023-05-08

**Authors:** Elias Barbosa de Oliveira, Tatiane Xavier, Regina Celia Gollner Zeitoune, Joanir Pereira Passos, Bruno Rafael de Oliveira, Ana Rita Alves Ferreira

**Affiliations:** I Universidade do Estado do Rio de Janeiro. Rio de Janeiro, Rio de Janeiro, Brazil; II Universidade Federal do Rio de Janeiro. Rio de Janeiro, Rio de Janeiro, Brazil; III Universidade Federal do Estado do Rio de Janeiro. Rio de Janeiro, Rio de Janeiro, Brazil; IV Instituto Nacional do Câncer. Rio de Janeiro, Rio de Janeiro, Brazil

**Keywords:** Nursing, Surgery Department, Hospital, Human Resources Management, Worker’s Health, Occupational Risk, Enfermería, Centro Quirúrgico Hospitalario, Gestión de Recursos Humanos, Salud del Trabajador, Riesgos Laborales, Enfermagem, Centro Cirúrgico Hospitalar, Administração de Recursos Humanos, Saúde do Trabalhador, Riscos Ocupacionais

## Abstract

**Objectives:**

to analyze the implications of precarious work for the organization of work and for the health of nursing professionals in a surgical center.

**Methods:**

qualitative, descriptive study in which the interview technique was applied on 30 nursing professionals from a surgical center in a university hospital located in the Southeast region of Brazil. The project was approved by the research ethics committee. Thematic content analysis was applied in the categorization of speeches.

**Results:**

precarious work in the surgical center negatively affects the organization of work due to staff turnover, loss of skilled talent, and the need for continuous training of temporary workers. It also affects the quality of care, leading to risks to patient safety and workers’ health.**Final Considerations:** it is important to make work conditions less precarious in order to minimize staff turnover and promote the quality of the service offered and the health of the worker.

## RESUMO

**Objetivos:**

analisar as implicações do trabalho precário para a organização do trabalho e para a saúde dos profissionais de enfermagem em centro cirúrgico.

**Métodos:**

estudo qualitativo, descritivo no qual se utilizou a técnica de entrevista com 30 profissionais de enfermagem de centro cirúrgico em um hospital universitário situado em um município da região sudeste, Brasil. Projeto aprovado por comitê de ética e pesquisa. Na categorização dos depoimentos aplicou-se a análise de conteúdo temática.

**Resultados:**

a precarização em centro cirúrgico interfere negativamente na organização do trabalho devido a rotatividade de pessoal, a fuga de capital intelectual e a necessidade de treinamento contínuo dos trabalhadores temporários. Há interferência na qualidade da assistência com riscos para a segurança dos pacientes e a saúde dos trabalhadores.

**Considerações Finais:**

ratifica-se a relevância da desprecarização do trabalho no intuito de minimizar a rotatividade dos profissionais, promover a qualidade do serviço ofertado e a saúde do trabalhador.

## RESUMEN

**Objetivos:**

analizar las implicaciones del trabajo precario para la organización laboral y la salud de los profesionales de enfermería en un centro quirúrgico.

**Métodos:**

se trata de un estudio cualitativo, descriptivo, llevado a cabo mediante entrevista con 30 profesionales de enfermería en un centro quirúrgico de un municipio de la región sureste de Brasil. El proyecto fue aprobado por el comité de ética e investigación. En la categorización de las declaraciones, se aplicó el análisis de contenido temático.

**Resultados:**

la precarización en centros quirúrgicos interfiere negativamente en la organización del trabajo debido a la rotación de personal, la fuga de capital intelectual y la necesidad de capacitación continua de los trabajadores temporarios. Existe una interferencia en la calidad de la atención con riesgos para la seguridad de los pacientes y la salud de los trabajadores.

**Consideraciones Finales:**

se ratifica la importancia de apuntalar el trabajo con el intuito de minimizar la rotación laboral y promover la calidad del servicio brindado y la salud del trabajador.

## INTRODUCTION

Hospitals treat patients with different clinical and/or surgical conditions. At some stage of treatment, these patients may need procedures that must be performed in surgical centers (SC), which must have a set of areas and facilities that allow safe anesthetic and surgical procedures for the patient and for the team.As it is a complex and diurnal sector, the service capacity depends on detailed planning of human and material resources done in the 24 hours before the surgeries. Therefore, professionals who work in SC must be in constant contact with the other hospital units, to communicate about unpredicted events that can lead to delays and suspension of surgeries, which increase the social and financial costs of the institution^(1-2)^.

For the effective running of operating rooms, the number of nursing staff must be in accordance with the classification of surgeries, the hours of assistance must be in line with the complexity of each surgery, and waiting time and cleaning of the rooms must be appropriate^(3)^. Another aspect involved in SC care is the knowledge, technological mastery and professional expertiserequired to perform aseptic procedures in the preintraand postoperative phases (anesthesia recovery). These activities should prioritize the safety, comfort and well-being of patients, considering the risks of complications due to the use of anesthetics, contamination of the surgical site, falls and burns^(4)^.

In the surgical planning, the nurse manager must be concerned with qualitative and quantitative aspects of human resources, aiming to meet the real needs of the patient and to ensure quality and safety of care. This is a dynamic process that involves the classification of patients, surgical demands, working hours, shifts, proportion of workers per sector, and number of rooms^(5)^. However, the precariousness of work resulting from new work models and flexibilization of work relationships has led to a reduction in the staff of university hospitals, with serious implications for the continuity of care, teaching and extension activities^(6)^. Supported by the Fiscal Responsibility Law (Complementary Law nº 11/2000) and considering the absence of new public tenders to keep the services operational, hospitals have adopted other strategies for hiring personnel through informal contracts (precariousness), which have led to the disorganization of work processes and impaired the quality of services^(7)^.

Precarious work is considered a result of deregulation of work, lack of social protection (full enjoyment of labor and social security rights) and loss of benefits guaranteed by the Constitution. This modality of work in the health sector has been associated with precarious working conditions, longer working hours, increased suffering, discontinuity of work projects, difficulty to retain qualified professionals, high demand for productivity, multi-skilling, and non-compliance of norms related to worker safety^(8-9)^.

Studies on nursing work in surgical centers describe that this environment is beset with problems that are closely related to the issue of precarious work, such as deficit of human and material resources and inappropriate communication between the sector and the hospital complex. These problems have repercussions on the work process, on quality of life at work and on the health of these professionals^(2,4-5)^. Considering the research gap on precarious work in surgical centers and its implications for the organization of work and the health of workers, the present study seeks to address these issues and contribute to the reflection on how precarious work can affect workers’ health and compromise the quality of the care provided by nursing workers and other teams.

To aggravate the situation, the labor reform created by Law no. 13,467, published in a time when work was already precarious, has escalated the relaxation of norms that protected the health of the workers, for example, by releasing the government from the obligation of conducting a prior inspection of work environments with any degree of unhealthiness (minimum, medium and maximum). Currently, due to changes in the CLT, the need for a prior license from the government to start an employment contract is now valid only for the maximum degree of unhealthiness, which leads to the exposure of nursing professionals to occupational risks and increases accidents and errors during patient care^(10)^.

Faced with this work reality, the question that emerges is: what are the implications of precarious work for the organization of work in a surgical center? How does precarious work in the surgical center affect the health of nursing workers?

## OBJECTIVES

To analyze the implications of precarious work for the organization of work and for the health of nursing professionals in a surgical center.

## METHODS

### Ethical aspects

The ethical precepts of research with human beings established in Resolution 466/12 were followed. The project was approved by the Research Ethics Committee of the institution where the study was carried out. Data were collected after the participants signed the Informed Consent Form. To preserve the anonymity of the respondents, the following codes were used in the transcription of the statements: letter I (Interviewee), followed by the abbreviation of the professional category (N=Nurse and NT=Nursing Technician), and a number referring to the order in which the interviews were carried out (N1, NT2...).

### Type of study

Descriptive qualitative study whose object refers to people’s perceptions, beliefs and values related to the world, their experiences and/or interpretations, applied to the study of history, relationships and representations^(11)^. The theoretical and methodological framework chosen was precarious work, based on the restructuring of production arising from Toyotism, which led to macrostructural changes in all productive sectors, including the health sector. In the social aspect of work, the problems observed include the reduction of State interference in the economy, reduction of legal protection of labor relationships, fragmentation of work, decline of unions, among others^(6-10)^.

The study setting was the surgical center of a large public university hospital in the Southeast region, recognized for its excellence in the areas of assistance, research and teaching. The sector has a capacity of 16 rooms and has, on average, more than 3,000 surgeries per month, in the most diverse specialties such as: general, pediatric, urological, cardiac, gynecological, vascular, proctological, plastic, neurological, ophthalmological, otorhinolaryngological, and others. Even though the hospital unit does not have an emergency service, the sector remains operational 24 hours a day for in-hospital care for urgent surgeries.

### Data source

The study included 30 nursing professionals (five nurses and 25 nursing technicians) from the permanent staff, who had been working in the surgical center for at least one year. Workers who were on vacation, on medical leave or any other leave during the data collection period were excluded.

### Data collection and organization

Data was collected from May to August 2016 through a semi-structured interview, which used an instrument to record sociodemographic data and a script elaborated by the authors, with four open questions about the object of study (What do you about hiring temporary staff for the surgical center? What factors contributed to hiring temporary staff for the surgical center? What are the repercussions of hiring temporary staff for the service? What are the implications of hiring temporary staff for workers’ health?). With the objective of improving the script and the interviews, a pilot test was carried out with two nursing professionals (a nurse and a nursing technician) who worked in another surgical center. After transcribing the data and discussing it with the collaborators, a new question was added to the script, and the material obtained was not part of the study. The interviews were conducted by a nurse from the graduate program (master’s degree), who introduced herself to the head of the unit and presented the purpose and objectives of the study. After obtaining the schedule of the service, the professionals were contacted for selection, invitation and scheduling. In a second moment, the person responsible for data collection introduced herself to the workers, re-explaining the objectives and presenting the authorization to enter the field. Data were collected individually, after obtaining the participants’ signature in the Informed Consent Form. The interviews were carried out in a private place that provided comfort, security and anonymity to the participants. The audio of the interviews was recorded on an MP3 recorder and later transcribed. Each interview lasted around 40 minutes and the transcription was presented to the participants, who were able to read and agree with the content of the transcripts. As the interviews were collected and transcribed and content analysis techniques were applied to the data to organize the categories and subcategories, saturation of data was observed^(11)^, meaning that new statements did not add new knowledge about precarious work in the surgical center when considering the proposed objectives.

### Data analysis

Thematic content analysis was applied in the elaboration of the categories emerging from the data^(12)^. A set of techniques was used to replicate and validate inferences through specialized and scientific procedures. At first, pre-analysis was carried out (transcription of the interviews and organization of the data being produced as the interviews were carried out, based on the scripts). Next, the material was explored to identify the recording units (in-depth reading with the objective of identifying points of convergence or divergence in the statements about the object of study). In this phase, the significant recording units (thematic units) were counted according to repetition of statements or units of meaning, homogeneity (convergence of statements) and representativeness in relation to the object and objectives of the study. At the end of this process, the following categories were elaborated: a) precariousness as an intervening factor in the organization of work in the surgical center (207 RU), with the subcategories: loss of skilled talent (85RU); staff turnover (67RU) and training / qualification of temporary workers (55RU); b) workers’ health in the context of precarious work in a surgical center (164 RU) and the subcategories: lack of labor rights (94 RU) and lack of social protection (70 RU).The discussions were based on precarious work in the health sector and in the nursing area, considering that no studies on precarious work in the surgical center were found.

## RESULTS

The study included 30 nursing workers who were mostly female (n=22), did not live with a partner (n=18), were over 46 years old (n=20), and had a family income greater than five minimum wages (n=18). Most participants had been working in the surgical center for more than 5 years (n=23), worked 30 hours a week at the institution in shifts (n=29) and worked over 60 hours a week (n=16) when considering other employment relationships.

The categories resulting from the content analysis are presented and discussed below:

### Precariousness as an intervening factor in the organization of work in a surgical center

Surgical procedures involve a series of technical and assistance measures, with professionals of different areas. For the surgeries to occur as planned in the surgical map, the staff involved must have knowledge and experience and be familiar with the routine of the sector. It is a complex work process that involves preparing the rooms, checking the entire surgical material, welcoming patients and performing several procedures that pose risks for patient safety. Therefore, planning the surgeries and organizing the entire work process are essential steps for performing procedures within the estimated time with no intercurrence that can harm the functioning and productivity of the service and the quality of care.

However, as reported by the participants, the lack of permanent positions calls to hire personnel to keep the service operational was one of the factors that contributed to precarious work in the surgical center. Among the problems that affect the organization of work, the following were pointed out: exhaustion of the teams due to the lack of knowledge of new temporary workers about the sector’s routine and staff turnover with the consequent loss of skilled talent, considering that the knowledge and skills acquired by temporary workers during in-service training are not always used in favor of the smooth running of the service due to termination or breach of contract.

*Most of the employees were retiring, getting sick and we did not have new permanent positions. So it was necessary to hire temporary staff.* (NT7)

*You need to restock human resources, also considering the increase in the service capacity, so we can handle the number of surgeries.* (N29)

*The temporary contract, I believe, brings physical and mental exhaustion for everyone, because the people who arrive do not necessarily know the routine.* (NT28)

*You can’t make progress in the training of that professional* [referring to the temporary worker]*. You see that the professionals that you are always training will not be used.* (NT1)

Other aspects pointed out by the participants in relation to the work carried out by temporary professionals were: the concern with the image of the sector, the safety of the procedures, and the exposure of patients to risks due the need for training and supervision of these professionals who do not master the work process. Problems related to the evaluation and monitoring of temporary workers due to a shortage of nursing staff were also identified, as reported:

*The risk for the sector and for the unit is very high, if you consider the safety of the procedures. It is a situation that constantly exposes the patient and the unit to risk, because you have workers being trained all the time.* (N3)

*We also want quality assistance, and to have that, we need training. Training is necessary to improve the service we provide to the population.* (NT6)

*We don’t have enough nurses to monitor the quality of care. Today, we can’t assess the quality of care provided with this group of workers.* (N29)

### Worker’s health in the context of precarious work in a surgical center

Although the institution chosen as study setting has an Occupational Health Service (SSO), medical monitoring and periodic examinations of temporary professionals are not within the competence of the SSO. This is because temporary workers are not governed by the Consolidation of Labor Laws (CLT) and, therefore, do not have labor rights related to health and safety at work. As reported by the participants, temporary workers were vulnerable due to lack of labor protection, especially in cases of accidents and/or illness, when they had to assume the social and financial burden of treatment and/or had to resort to the SUS.

*They are working here and they don’t have the same rights as us! They can’t get sick, they can’t do this, they can’t do that. They can’t take a leave, they can’t take a vacation.* (NT15)

*The temporary worker is always jeopardized, because they don’t have work benefits! They don’t have a bond. If temporary workers are injured, or have an accident at work, how will they be treated?* (NT26)

*There are temporary workers who are afraid to take a sick leave. This might even aggravate their condition. They avoid being absent, for fear of leaving to see a doctor and losing their job here.* (NT10)

## DISCUSSION

The debate about human resources in nursing involves political and economic issues that affect all planning, because, even with the use of instruments for calculating the need for personnel, services have a shortage of workers in relation to their real needs. In nursing, problems such as breach of contract, unexcused absences, absenteeism, sick leaves and other absences affect the work process and the provision of services, due to difficulties in replacing personnel. This situation is a reflection of the implementation and stabilization of neoliberal policies that brought important changes to Brazilian labor and social security legislation, relaxing the regulations on labor relationships^(6)^. For the State, this is an advantageous strategy, due to lower labor costs, but it comes with serious consequences for the organization of health services. Supported by the Fiscal Responsibility Law (Complementary Law nº 11/2000), the flexibility of hiring temporary staff provides greater freedom to the employer in the admission or dismissal of workers; however, its consequences must be examined with caution, especially in the health sector, where workers deal with human lives^(13-14)^.

The results show that this situation of precarious work is reinforced as professionals accept its effects, remaining at work and being bound to the reality of lack of social protection and absence of rights guaranteed in the CLT, among other stressful situations. This reality corroborates a study on precarious work and work overload among nurses who, in addition to managing the absences of permanent workers, have to deal the turnover of temporary professionals, who, due to instability, terminate the contract to look for better opportunities or are dismissed by the institution^(15-16)^. The admissions and dismissal of personnel in the nursing services incur in financial and social costs to the institutions due to the process of selection, training and supervision. The solution to some factors that affect the quality of services lies in the investment in policies for employee retention, which can minimize contract terminations and loss of skilled talent^(7)^. Staff turnover in public services is associated with the employment instability of precarious work, in which the retraction of the State’s role as market regulator and the lack of social protection of workers can be observed. This issue affects all professionals, regardless of the statute which governs their employment, and has led to deterioration of working conditions, loss of bargaining power of unions, and risks to workers’ health^(8-10)^

Another relevant aspect in the discussions about precarious work in nursing is that the surgical center, a restricted and highly complex sector, requires professionals to use specific knowledge and skills to make quick decisions, which involve risk for patients, as they are undergoing invasive procedures with the possibility of iatrogenesis and complications^(2,4-5)^. Therefore, regardless of contract or training modality, professionals’ knowledge and practices must be up to date with scientific and technological advances in the field, and the institution is responsible for their training and supervision, aiming at safety. Performing procedures, especially invasive ones, without the proper knowledge and/or skills makes professionals feel insecure and apprehensive, and may be another factor that generates stress in surgical center, in circumstances where there are risks for patients and for professional practice^(17)^. On the other hand, a study carried out in 159 surgical centers found that nursing workers were at low risk for pathogenic stress in work contexts that enabled cooperation, freedom of expression and harmonious coexistence, factors that contributed to feelings of usefulness and accomplishment^(18)^.

In a sector of high complexity such as SC, precarious work conditions expose professionals to ethical dilemmas that affect their subjectivity in the face of technical limitations and the need for improvement. The high emotional and social burden imposed by the responsibilities of temporary professionals, considering the nature of the work performed and the risks inherent to this practice, must also be considered^(16)^. A study addressing the suffering of nursing professionals sued for errors in procedures, identified that the worker is not always able to intervene in errors, precisely because of inadequate working conditions related to the shortage of human and material resources, which increase workloads and contribute to the risk of incidents. Lack of support from the institution and from professional associations when these professionals have to face justice leads to feelings of injustice, revolt and impotence, with reports of abandonment of the profession^(19)^.

Strategies to prevent errors and/or accidents in SC can be adopted through training that allows the individual to know the entire work process, the institution’s policy and other relevant information. There must be a performance evaluation system with well-defined objectives, and methods consistent with institutional policy and philosophy must be applied^(4-5)^. A high staff turnover requires investment of time and human resources to train professionals who will not necessarily stay in the job; this characteristic of precarious work destabilizes the functioning of services and personnel management processes^(7,16)^. It should be noted that job instability can reduce professionals’ commitment to qualification, since, in current management models, the workers are responsible for their own training, and institutions do not offer financial incentives or reduce workloads for professionals who participate in refresher courses^(20)^.

Health workers on temporary contracts not only have the lowest wages, work longer hours, and have their labor rights provided for by law denied, but are also more exposed to risks of accidents, illness and death due to inadequate safety and health conditions at work, in the face of new occupational risks related to technological and therapeutic advances^(15,21-22)^. As for the physical facilities, SC are restricted areas, with artificial ventilation and lighting, where workers coexist with the risk of accidents and exposure to biological, chemical, ionizing and ergonomic material that can cause damage to health, even if a prevention culture is in place^(17-18)^.Among the ergonomic risks, it is observed that, due to the performance of surgical procedures and other care activities, nursing professionals maintain static postures, remaining standing and/or sitting for a long time. This can lead to musculoskeletal problems, varicose veins, tiredness, headaches and fatigue^(23-24)^. There are also organizational issues related to the demand for high productivity combined with eventual understaffing, which results in overload and work intensification in the surgical center, leading to suffering and exhaustion^(25)^.

In precarious work, organizations that seek greater productivity at all costs have provided their workers with very strenuous work conditions, through work intensification strategies such as establishing goals that are not always achievable, extending the working day and multi-skilling^(21)^. These strategies are associated with fear-based management, with subtle forms of abuse of power and perpetuation of a work environment that is harmful to health and in which organizations do not always provide clarification on the risks and collective measures for protection and prevention against accidents^(22)^.

The participants reported that, as temporary workers are not entitled to leave to treat health problems, they avoid missing work for fear of being punished or dismissed. This leads to presenteeism, a situation in which the individual remains at work while sick. This can pose risks to the health of this professional, as acute and/or chronic conditions may aggravate. In addition, it is necessary to consider the possible damage to the quality of the service and conflicts in interpersonal relationships, as the team may struggle with work overload while trying to meet the demands generated by the low productivity of that professional^(26)^.

The deregulation of social, social security and labor rights, with the consent of the State, allows unsafe work conditions that harm professional dignity and citizenship. In addition, the weakening of unions leads to the relaxation of labor rights, with the endorsement of organizations and acceptance of professionals who, due to contract differences and different interests, do not offer resistance. Workers are alienated from the struggle for better working conditions and quality of life, leading them to accept conditions of social insecurity and helplessness so that, through precarious work, they can have their means of subsistence^(8-9)^.

### Study limitations

As this is a qualitative study conducted with part of the permanent staff, its limitations are related to the method, the number of participants and the fact it was carried out in a single institution. The lack of studies on precarious work in the surgical center was a limitation for comparison and discussion of the findings.

### Contributions to Nursing

As the SC is a highly complex sector, where workers and users are exposed to all kinds of risks, the relevance of the study is ratified, as it contributes to discussion and reflection on the improvement of work conditions, in line with the *Política Nacional Interinstitucional de Desprecarização do Trabalho no SUS*. By ensuring the labor rights provided for in the CLT, the professionals are valued and turnover and loss of skilled talent are reduced, with positive effects on the organization of work, the quality of care and the health of nursing workers.

## FINAL CONSIDERATIONS

Precarious work in the surgical center negatively affects the organization of work due to staff turnover, loss of skilled talent, and the need for continuous training of temporary workers. It also affects the quality of care, leading to risks to patient safety and workers’ health. Therefore, it is important to make work conditions less precarious in order to minimize staff turnover and promote the quality of the service offered and the health of the worker.
